# GCN5L1 Aggravates Postherpetic Neuralgia Through Regulating Microglial Mitochondrial Fission–Fusion Homeostasis

**DOI:** 10.1111/jcmm.70861

**Published:** 2025-09-23

**Authors:** Wang Li, Xin Cao, Shenghan Wang, Xuedong Jin, Hongqian Wang

**Affiliations:** ^1^ Department of Anesthesiology Shandong Provincial Hospital Affiliated to Shandong First Medical University Jinan Shandong China; ^2^ Department of Anesthesiology Jinan Seventh People's Hospital Jinan Shandong China

**Keywords:** GCN5L1, microglia, mitochondrial fission–fusion, neuroinflammation, postherpetic neuralgia

## Abstract

Postherpetic neuralgia (PHN) is a debilitating chronic pain condition following varicella‐zoster virus (VZV) reactivation, characterised by persistent neuroinflammation. However, the intracellular mechanisms that drive microglial activation and sustained pain sensitisation remain poorly understood. Due to mice having no VZV infection receptor, herpes simplex virus type 1 (HSV‐1) infection is a well‐established PHN mice model. Here, we identified GCN5L1, a mitochondrial acetylation modulator, as a critical regulator of microglial mitochondrial dynamics and a key contributor to PHN pathogenesis. We found that GCN5L1 was markedly upregulated in the spinal dorsal horn after PHN, particularly located in microglia. Microglial *Gcn5l1* deficiency attenuated HSV‐1‐induced neuroinflammatory responses and alleviated mechanical allodynia, whereas *Gcn5l1* overexpression exacerbated neuroinflammatory responses both in vivo and in vitro. Mechanistically, GCN5L1 promoted mitochondrial fission and impaired oxidative metabolism by enhancing DRP1 acetylation, without altering the expression of canonical fission–fusion regulators. Restoration of mitochondrial fission using MFI8 intrathecally reversed the anti‐inflammatory and analgesic effects of *Gcn5l1* deficiency, confirming that GCN5L1 mediated pain sensitisation through mitochondrial fission–fusion in PHN. Finally, inhibiting GCN5L1 by AAV‐shGCN5L1 intrathecally suppressed neuroinflammation and mechanical allodynia in PHN mice. These findings uncovered that GCN5L1 aggravated neuroinflammation and PHN through regulating microglial mitochondrial fission–fusion homeostasis, offering new insights and potential feasibility in clinical translation for PHN management.

## 1 Introduction

Postherpetic neuralgia (PHN) is a debilitating chronic pain syndrome that persists long after the resolution of herpes zoster rash, severely impairing the quality of life in affected individuals. PHN is primarily triggered by the reactivation of latent varicella zoster virus (VZV) in the dorsal root ganglia and spinal cord, leading to persistent sensory dysfunction [1]. Accumulating evidence indicates that the transition from acute infection to chronic pain is not merely a consequence of neuronal injury but is strongly driven by sustained neuroinflammation within the central nervous system [2, 3]. Among the central immune cells, microglia, resident macrophage‐like cells in the spinal cord, have emerged as pivotal mediators of neuroinflammatory responses associated with PHN. Upon VZV reactivation, microglia are activated and undergo a pro‐inflammatory transformation, releasing cytokines and reactive species that sensitise pain pathways [4]. However, the molecular mechanisms that govern microglial activation and their contribution to PHN pathogenesis remain incompletely understood, underscoring the need for deeper mechanistic insights.

Although VZV is the etiological agent of PHN in humans, its strict species specificity limits the development of reliable animal models. Herpes simplex virus type 1 (HSV‐1) has been widely adopted as an alternative in experimental models, given its close phylogenetic relationship with VZV [5]. Beyond responding to exogenous insults, microglial activation is tightly regulated by intrinsic metabolic cues, particularly mitochondrial function. Mitochondrial dynamics, namely the continuous processes of fission and fusion, are central to preserving mitochondrial homeostasis and shaping immune responses in microglia. Disruption of this delicate balance profoundly alters the metabolic and inflammatory state of these resident immune cells in the central nervous system [6]. Excessive mitochondrial fission leads to the release of mitochondrial DNA (mtDNA) into the cytoplasm, a potent activator of the cGAS‐STING pathway that amplifies neuroinflammatory signalling. Concurrently, mitochondrial reactive oxygen species (mtROS) accumulate under conditions of aberrant fission, exacerbating oxidative stress and further potentiating microglial activation [7]. Moreover, imbalanced mitochondrial dynamics contribute to immunometabolic reprogramming, suppressing oxidative phosphorylation while enhancing glycolysis, a metabolic shift that sustains the pro‐inflammatory phenotype of activated microglia. These findings highlight mitochondrial dynamics as a pivotal node linking cellular metabolism to innate immune signalling within the central nervous system, offering mechanistic insight into chronic neuroinflammation in disorders such as PHN [8].

Mitochondrial fission and fusion are orchestrated by a tightly regulated set of GTPase proteins that determine the structural and functional integrity of the mitochondrial network. Dynamin‐related protein 1 (DRP1) is the central executor of mitochondrial fission; it is recruited from the cytosol to the outer mitochondrial membrane, where it oligomerizes and constricts mitochondria to promote division. This recruitment is facilitated by adaptor proteins including fission protein 1 (FIS1) and mitochondrial fission factor (MFF), which anchor DRP1 to the outer mitochondrial membrane [9]. In contrast, mitochondrial fusion is mediated by mitofusin 1 and 2 (MFN1/2) for outer mitochondrial membrane tethering and merging, and optic atrophy protein 1 (OPA1) for inner mitochondrial membrane remodelling [10]. Under physiological conditions, a dynamic balance between fission and fusion is essential to maintain mitochondrial morphology, bioenergetic capacity and quality control. Disruption of this equilibrium results in mitochondrial fragmentation, triggering downstream consequences such as cytosolic release of mtDNA, elevated mtROS production, and a metabolic shift from oxidative phosphorylation to glycolysis, thereby reinforcing a pro‐inflammatory microglial phenotype. These regulatory proteins collectively serve as critical nodes in linking mitochondrial dynamics to innate immune signalling and are thus potential effectors or targets of upstream modulators [11].

General control of amino acid synthesis 5 like 1 (GCN5L1) is a mitochondrial‐localised protein known to mediate lysine acetylation of multiple mitochondrial enzymes, thereby modulating metabolic flux and organelle function [12]. While GCN5L1 has been implicated in metabolic regulation under physiological and pathological conditions, its role in the central nervous system, particularly in the context of neuroinflammatory pain disorders such as PHN, remains largely unexplored. Recent evidence has hinted at mitochondrial protein acetylation as a pivotal modulator of mitochondrial dynamics, raising the possibility that GCN5L1 may serve as a functional link between metabolic disturbance and immune activation [13, 14]. In this study, we investigated the expression and functional significance of GCN5L1 in the spinal microglia of a murine PHN model. We specifically hypothesised that GCN5L1 promoted microglial neuroinflammation by enhancing the acetylation and activation of DRP1, thereby facilitating mitochondrial fission and the acquisition of a pro‐inflammatory phenotype. Furthermore, we evaluated whether targeting GCN5L1 could mitigate PHN‐related neuroinflammation and pain hypersensitivity, thus providing a novel therapeutic strategy for this debilitating condition.

## 2 Materials and Methods

### 2.1 Mice

Male C57BL/6J mice weighing 20–25 g were procured from Shandong First Medical University (Jinan, China). To generate microglia‐specific *Gcn5l1* conditional knockout mice (*Tmem119* ^*CreERT2*^ *Gcn5l1* ^*flox/flox*^, referred to as *Gcn5l1*‐CKO), we utilised two genetically engineered mouse strains: *Tmem119‐CreERT2* and *Gcn5l1‐flox/flox*. The *Tmem119‐CreERT2* line expresses a tamoxifen‐inducible *Cre* recombinase under the control of the microglia‐specific *Tmem119* promoter, enabling temporal and cell‐type‐specific gene recombination. The *Gcn5l1‐flox/flox* mice carry loxP sites flanking critical exons of the *Gcn5l1* gene, allowing for conditional deletion upon *Cre* activation. To obtain *Gcn5l1*‐CKO mice, male *Tmem119‐CreERT2* mice were crossed with female *Gcn5l1‐flox/flox* mice. Offspring carrying both the *Tmem119‐CreERT2* transgene and homozygous *Gcn5l1* floxed alleles were identified as conditional knockouts. Littermates lacking the *Cre* transgene were used as wild‐type controls (referred to as *Gcn5l1*‐WT). For inducible recombination, tamoxifen (Sigma‐Aldrich, T5648) was dissolved in corn oil (20 mg/mL) and administered intraperitoneally at 100 mg/kg daily for 5 consecutive days. All mice were allowed to rest for at least 14 days post‐injection before any further experimental procedures to ensure complete recombination and clearance of tamoxifen. All animals were housed in a specific‐pathogen‐free facility under controlled environmental conditions with ad libitum access to food and water. All experimental procedures complied with institutional and national guidelines for animal care and use.

### 2.2 Reagents

MFI8 (MedChemExpress, HY‐150031) was used to enhance mitochondrial fission and inhibit fusion by intrathecal injection (0.5 μg/5 μL) for in vivo studies. Antibodies used in this study were: GCN5L1 antibody (Biodragon, BD‐PN6046), p‐p65 antibody (Abcam, ab53489), NLRP3 antibody (Abcam, ab263899), FIS1 antibody (Beyotime, AG5005), DRP1 antibody (Beyotime, AF5791), MFF antibody (Biorbyt, orb1257634), MFN1 antibody (Biorbyt, orb1318457), MFN2 antibody (Abcam, ab124773), OPA1 antibody (Abcam, ab157457), Acetylated‐lysine antibody (Cell Signalling Technology, 9441), anti‐β‐actin antibody (Cell Signalling Technology, 4967). TNF‐α Mouse ELISA Kit (Invitrogen, 88‐7324‐88), IL‐1β Mouse ELISA Kit (Invitrogen, 900‐M47), IL‐6 (Invitrogen, 88‐7064‐88) were used to detect the protein levels of inflammatory cytokines. MitoSOX Red (Invitrogen, M36008) was used to detect mtROS.

### 2.3 PHN Model Establishment

PHN mouse model was generated via cutaneous infection with HSV‐1 (strain 7401H), following previously established protocols [5]. Mice were first shaved and depilated on the lower back, flanks and hindlimbs. Three days later, the right hindlimb was lightly abraded using a 27‐gauge needle, after which 10 μL of HSV‐1 suspension (10^6^ PFU) was applied to the site. For control animals, HSV‐1 was heat‐inactivated by incubation at 60°C for 60 min prior to application. Skin lesion severity was scored on a 0–10 scale: 0 denoted no visible lesions; 2 represented one or two vesicles on the dorsal skin; 4 indicated multiple vesicles localised to the inoculation area; 6 signified mild herpes zoster‐like rash; 8 reflected distinct zosteriform lesions or hindpaw swelling; and 10 indicated severe vesicular eruptions resembling zoster.

### 2.4 Measurements of Mechanical Pain Thresholds

Mechanical sensitivity testing was conducted between 09:00 and 11:00 in a quiet environment. Mice were acclimated for 2 h in individual transparent Plexiglas chambers placed atop a wire mesh platform to allow access to the plantar surface. Mechanical allodynia was applied using calibrated von Frey filaments (0.6 and 2 g; North Coast Medical, USA) to the plantar surface. The 0.6 g filament was used to assess responses to low‐intensity mechanical input, while the 2 g filament tested sensitivity to higher‐force stimuli. The plantar surface was stimulated six times at one‐minute intervals. Behavioural responses were scored as follows: 0 for no response; 1 for withdrawal of the stimulated area; and 2 for flinching or licking. A pain sensitivity index was calculated using the formula: pain score (% of maximum) = (total score/12) × 100 [15, 16]. Mice typically developed herpes zoster‐like cutaneous lesions within 7–10 days following HSV‐1 inoculation. These lesions resolved with scarring by Day 14. Mice that continued to exhibit mechanical hypersensitivity after Day 14 were classified as having developed PHN.

### 2.5 Cultivation of Primary Microglia and BV‐2 Microglia Cells

Neonatal mice were euthanised under sterile conditions to enable the collection of lumbar spinal dorsal horn tissues. The dissected tissue was rinsed in ice‐cold PBS, finely chopped and enzymatically dissociated using 0.25% trypsin at 37°C for 10 min. Digestion was halted by supplementing with DMEM containing 10% FBS. The cell suspension was then gently triturated to achieve a single‐cell mixture and centrifuged to pellet the cells. Pellets were resuspended in 10% FBS/DMEM and seeded into poly‐L‐ornithine‐coated T‐25 flasks, followed by incubation at 37°C with 5% CO_2_. Medium changes were performed daily for 8 consecutive days. To separate microglia, cultures were agitated at 200 rpm for 5 min, allowing microglia to detach from the underlying astrocyte layer. The supernatant was collected, centrifuged and the resulting cell pellet was resuspended in fresh medium for downstream experiments. BV‐2 microglial cells were cultured under identical conditions (37°C, 5% CO_2_) in 10% FBS/DMEM, with media refreshed daily until cultures reached 80% confluency.

### 2.6 Isolation of Microglia From Spinal Dorsal Horn by Flow Cytometry

Microglia were isolated from the spinal dorsal horn (segments L3–L5) using fluorescence‐activated cell sorting (FACS). Briefly, spinal cords were rapidly dissected and the dorsal horn regions were microdissected, minced and enzymatically digested in a solution containing papain and DNase I at 37°C for 30 min. The resulting cell suspension was filtered through a 70 μm cell strainer and washed with cold PBS. Cells were resuspended in FACS buffer and stained with fluorophore‐conjugated antibodies against CD11b (APC) and CD45 (PE) for 30 min at 4°C in the dark. Dead cells were excluded using 7‐AAD staining. Microglia were identified and sorted as CD11b^+^CD45^int^ populations using a BD FACSAria II cell sorter. Sorted cells were immediately used for downstream assays including RNA and protein extraction.

### 2.7 Western Blotting

To analyse protein expression, tissues from the spinal dorsal horn and cultured microglia were collected. Proteins were extracted by sonicating the samples in ice‐cold RIPA lysis buffer and concentrations were determined using the BCA assay. Lysates were denatured by heating at 99°C for 10 min and then resolved on 10% SDS‐PAGE. Following electrophoresis, proteins were transferred onto PVDF membranes. Membranes were blocked with 5% BSA for 2 h at room temperature, then incubated overnight at 4°C with appropriate primary antibodies. After washing, membranes were incubated for 2 h with HRP‐conjugated secondary antibodies and protein bands were visualised using an enhanced chemiluminescence detection system.

### 2.8 Quantitative Polymerase Chain Reaction (Q‐PCR)

Total RNA was extracted from spinal dorsal horn tissues and cultured microglia using the TRIzol reagent, following the manufacturer's protocol. Complementary DNA (cDNA) was synthesised via reverse transcription using a commercially available kit. Q‐PCR was subsequently conducted on a QuantStudio 5 system (Applied Biosystems) with SYBR Green chemistry. Expression levels of target genes were quantified relative to β‐actin as the internal control, and fold changes were determined using the $2^{-∆∆C_{T}}$ method.

### 2.9 MitoSOX Red to Detect Mitochondria‐Specific ROS

mtROS levels were measured using MitoSOX Red according to the manufacturer's protocol. Microglial cells cultured on glass coverslips were incubated with 5 μM MitoSOX Red in serum‐free medium at 37°C for 15 min, protected from light. Following incubation, cells were gently washed three times with warm PBS to remove unbound dye. Fluorescent images were captured immediately using a confocal laser scanning microscope with excitation at 510 nm and emission collected at 580 nm. Image acquisition settings were kept constant across all samples. Quantification of mtROS levels was performed by measuring mean fluorescence intensity (MFI) using ImageJ software.

### 2.10 Transmission Electron Microscopy (TEM)

Mice were humanely euthanised and perfused transcardially with PBS followed by 0.25% glutaraldehyde. Lumbar spinal cord segments were rapidly dissected and immersed in 2.5% glutaraldehyde. Tissues were then sliced into 1 mm^3^ sections using a vibratome, post‐fixed in 1% osmium tetroxide for 1 h, and dehydrated through an ascending ethanol series. Samples were embedded in epoxy resin and polymerised at 80°C for 24 h. Ultrathin sections (60 nm) were cut with an ultramicrotome and stained with uranyl acetate and lead citrate to enhance contrast. Imaging was performed using a Thermo Scientific Talos F200S transmission electron microscope under standard TEM conditions to examine spinal cord ultrastructure.

### 2.11 Enzyme‐Linked Immunosorbent Assay (ELISA)

The protein levels of inflammatory cytokine were quantified using ELISA kits, following the manufacturer's protocols. Briefly, 100 μL of capture antibody was added to each microplate well and incubated overnight at 4°C. Wells were then washed three times with the provided wash buffer and blocked with 300 μL assay diluent for 1 h at room temperature. Standards and samples were prepared in appropriate dilutions, and 100 μL was added per well, followed by a 2‐h incubation at room temperature. After washing, 100 μL of detection antibody was added to each well and incubated for 1 h at room temperature. Wells were washed again before adding 100 μL of streptavidin‐HRP conjugate and incubated for 30 min. Colour development was initiated with 100 μL substrate solution in the dark until a visible blue colour appeared, then stopped by adding 100 μL stop solution. Absorbance was measured using a microplate reader at the specified wavelength, and protein concentrations were determined by comparison to a standard curve generated from known standards.

### 2.12 siRNA Construction and Transfection

Custom‐designed siRNAs targeting mouse *Gcn5l1* were synthesised by OBIO Biotechnology (Shanghai). BV‐2 cells were seeded into 24‐well plates at a density of 2 × 10^4^ cells/cm^2^, reaching 30%–50% confluence prior to transfection. For transfection, siRNAs and Lipofectamine 2000 were each diluted separately in Opti‐MEM, then mixed and added to the cells. The cells were incubated with this transfection mixture in antibiotic‐free DMEM containing 10% FBS for 6 h. Subsequently, the medium was replaced with fresh FBS/DMEM, and the cells were cultured for an additional 48 h. A scrambled siRNA sequence was used as a negative control.

### 2.13 Plasmid Construction and Cell Transfection

Plasmids encoding mouse *Gcn5l1* were custom synthesised by OBIO Biotechnology (Shanghai). A scrambled plasmid served as the negative control. BV‐2 cells were cultured in 24‐well plates until reaching approximately 80%–90% confluence. For transfection, plasmid DNA and Lipofectamine 2000 were separately diluted in Opti‐MEM, then combined and added to the cells. After transfection, cells were maintained in DMEM supplemented with 10% FBS for 48 h.

### 2.14 Intrathecal Injection of AAV‐shGCN5L1 for Microglial *Gcn5l1* Knockdown

To achieve targeted inhibition of *Gcn5l1* expression in spinal microglia of mice, we used adeno‐associated virus serotype 2/9 (AAV2/9) vectors carrying a short hairpin RNA (shRNA) against mouse *Gcn5l1*, driven by the microglia‐preferential CD68 promoter (AAV2/9‐CD68‐shGCN5L1, OBIO Biotechnology, Shanghai). Control animals received a non‐targeting scrambled shRNA under the same promoter (AAV2/9‐CD68‐shScramble). Mice were anaesthetised and placed in a prone position. A 30‐gauge Hamilton syringe was used to perform a lumbar intrathecal injection at the L4–L5 intervertebral space. A volume of 10 μL (1 × 10^12^ viral genomes/mL) was slowly injected per mouse. Following injection, animals were monitored until full recovery and housed under standard conditions. This promoter‐serotype combination has been validated in prior studies for preferential transduction of microglia in the central nervous system, and was selected to ensure cell‐type specificity of *Gcn5l1* knockdown in vivo [17, 18].

### 2.15 Measurement of Cytosolic mtDNA

Cytoplasmic mtDNA was quantified following a previously established protocol [19]. Cells were lysed in a mild buffer (50 mM HEPES at pH 7.4, 150 mM NaCl and 25 μg/mL digitonin) for 10 min at 4°C. The lysates were centrifuged at 980 *g* for 3 min to pellet intact cells, and this step was repeated three times. The resulting pellet was reserved for immunoblotting. The supernatant, containing cytosolic components, was carefully collected and subjected to a high‐speed centrifugation at 17,000 *g* for 10 min to eliminate mitochondrial contamination. DNA was then extracted from the clarified cytosolic fraction using the Cell/Tissue DNA Isolation Kit (Yeasen, 18700ES50). Relative mtDNA levels in the cytosol were quantified by Q‐PCR.

### 2.16 Extracellular Flux Assay

To assess glycolytic activity and mitochondrial respiration in microglial cells, extracellular flux analysis was conducted using the Seahorse XFe24 Analyser (Agilent Technologies, USA). Cells were seeded at 5 × 10^4^ cells per well in Seahorse XF24 microplates. Prior to measurement, cells were incubated for 1 h in a CO_2_‐free incubator at 37°C with XF assay medium supplemented with 10 mM glucose, 2 mM L‐glutamine and 1 mM sodium pyruvate. Oxygen consumption rate (OCR) was recorded at baseline and following sequential injections of oligomycin (to assess ATP‐linked respiration), FCCP (to determine maximal respiration) and rotenone/antimycin A (R/A, to measure non‐mitochondrial respiration). Extracellular acidification rate (ECAR) was measured starting with baseline readings, followed by injections of glucose (10 mM), oligomycin (1 μM) and 2‐deoxy‐glucose (2‐DG, 50 mM) to evaluate glycolytic flux and capacity.

### 2.17 Statistical Analysis

Statistical analyses were conducted using IBM SPSS Statistics version 23.0. Data are presented as mean ± SEM. Differences in mechanical pain thresholds over time among different groups were analysed using two‐way repeated measures ANOVA. Comparisons between two groups were performed using unpaired Student's *t*‐tests, while one‐way ANOVA followed by Dunnett's post hoc test was applied for multiple group comparisons. A *p*‐value less than 0.05 was considered statistically significant.

## 3 Results

### 3.1 Increased Microglial GCN5L1 Expression in PHN Promotes Neuroinflammation and Pain Sensitisation

**FIGURE 1:**
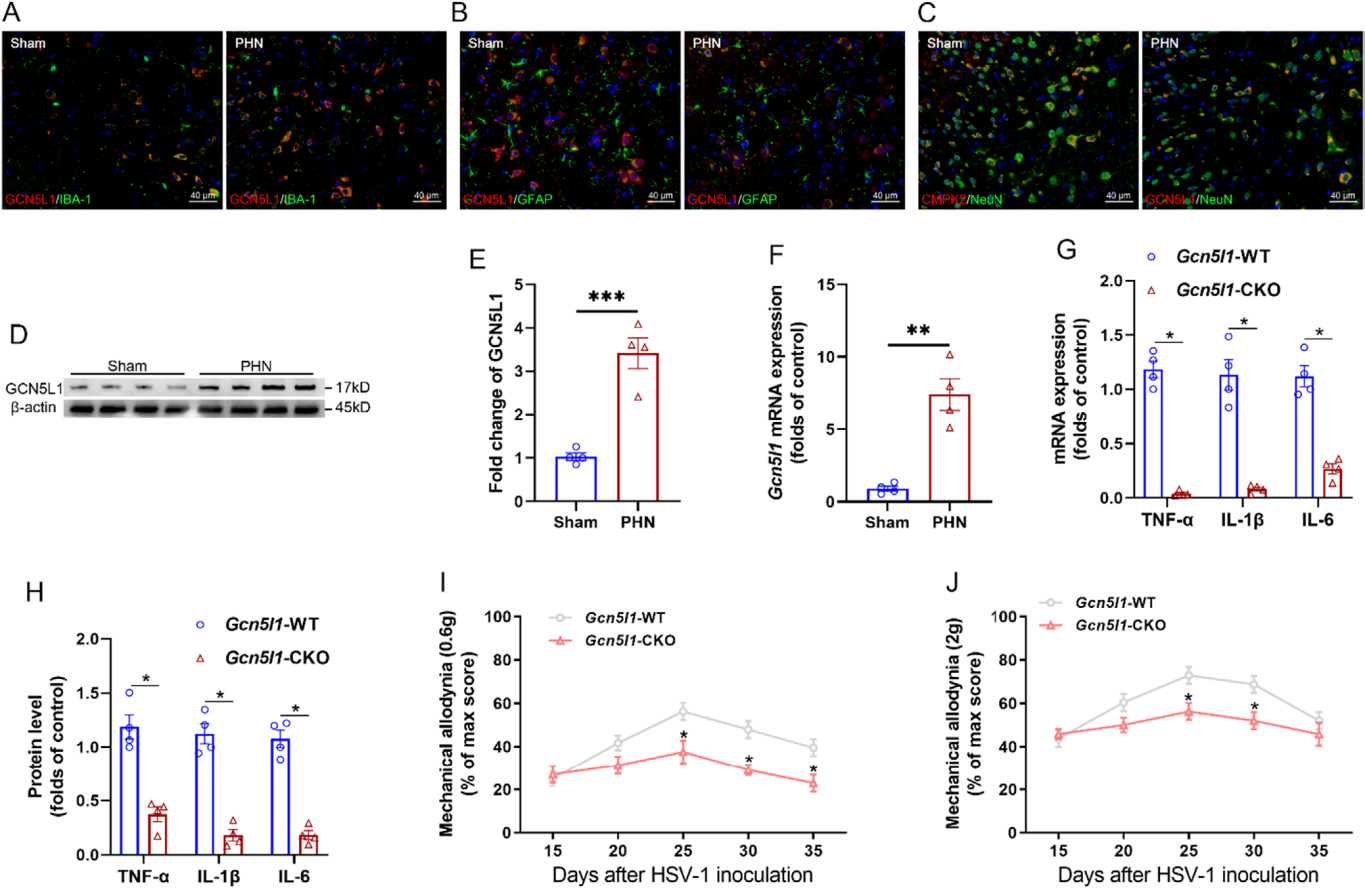
Increased microglial GCN5L1 expression promotes neuroinflammation and pain sensitisation in PHN. (A–C) Co‐expression of GCN5L1 with microglial marker IBA‐1 (A), astrocytic marker GFAP (B), and neuronal marker NeuN (C). (D, E) Western blot analysis (D) and quantification (E) of GCN5L1 protein levels in spinal microglia from PHN mice. (F) Transcriptional level of *Gcn5l1* in spinal microglia assessed by Q‐PCR. (G, H) The transcription (G) and protein (H) levels of TNF‐α, IL‐1β, and IL‐6 in spinal microglia of *Gcn5l1*‐WT and *Gcn5l1*‐CKO mice after PHN. (I, J) Mechanical allodynia of *Gcn5l1*‐WT and *Gcn5l1*‐CKO mice measured with 0.6 g (I) and 2.0 g (J) von Frey filaments in the PHN model. *n* = 4, *p < 0.05.

We first investigated the cellular localization and the expression profile of microglial GCN5L1 in the PHN mouse model. Immunofluorescence indicated an enhanced co‐localization of GCN5L1 with the microglial marker IBA‐1 after PHN (Figure 1A), while GCN5L1 did not show co‐localization with the astrocytic marker GFAP (Figure 1B). Crucially, its co‐localization with the neuronal marker NeuN remained unchanged (Figure 1C). Western blot analysis (Figure 1D,E) and Q‐PCR (Figure 1F) revealed a significant upregulation of GCN5L1 at both the protein and transcription levels in microglia isolated from the spinal dorsal horn following PHN. Given that neuroinflammation is a key pathological feature of PHN triggered by reactivation of HSV‐1 infection, we next assessed the functional role of microglial GCN5L1 in this context. We generated microglia‐specific *Gcn5l1* conditional knockout mice (*Gcn5l1*‐CKO) and control mice (*Gcn5l1*‐WT) and found that microglial *Gcn5l1* deficiency markedly reduced the transcription (Figure 1G) and protein (Figure 1H) levels of inflammatory cytokines (TNF‐α, IL‐1β and IL‐6) in PHN mice. Consistently, mechanical allodynia to 0.6 and 2 g von Frey filaments was significantly alleviated in *Gcn5l1*‐CKO mice compared to *Gcn5l1*‐WT mice with PHN (Figure 1I,J). These findings indicated that microglial GCN5L1 contributes to neuroinflammation and pain sensitization in the progression of PHN.

### 3.2 Microglial *Gcn5l1* Deficiency or Overexpression In Vitro Regulates HSV‐1‐Induced Inflammation

**FIGURE 2:**
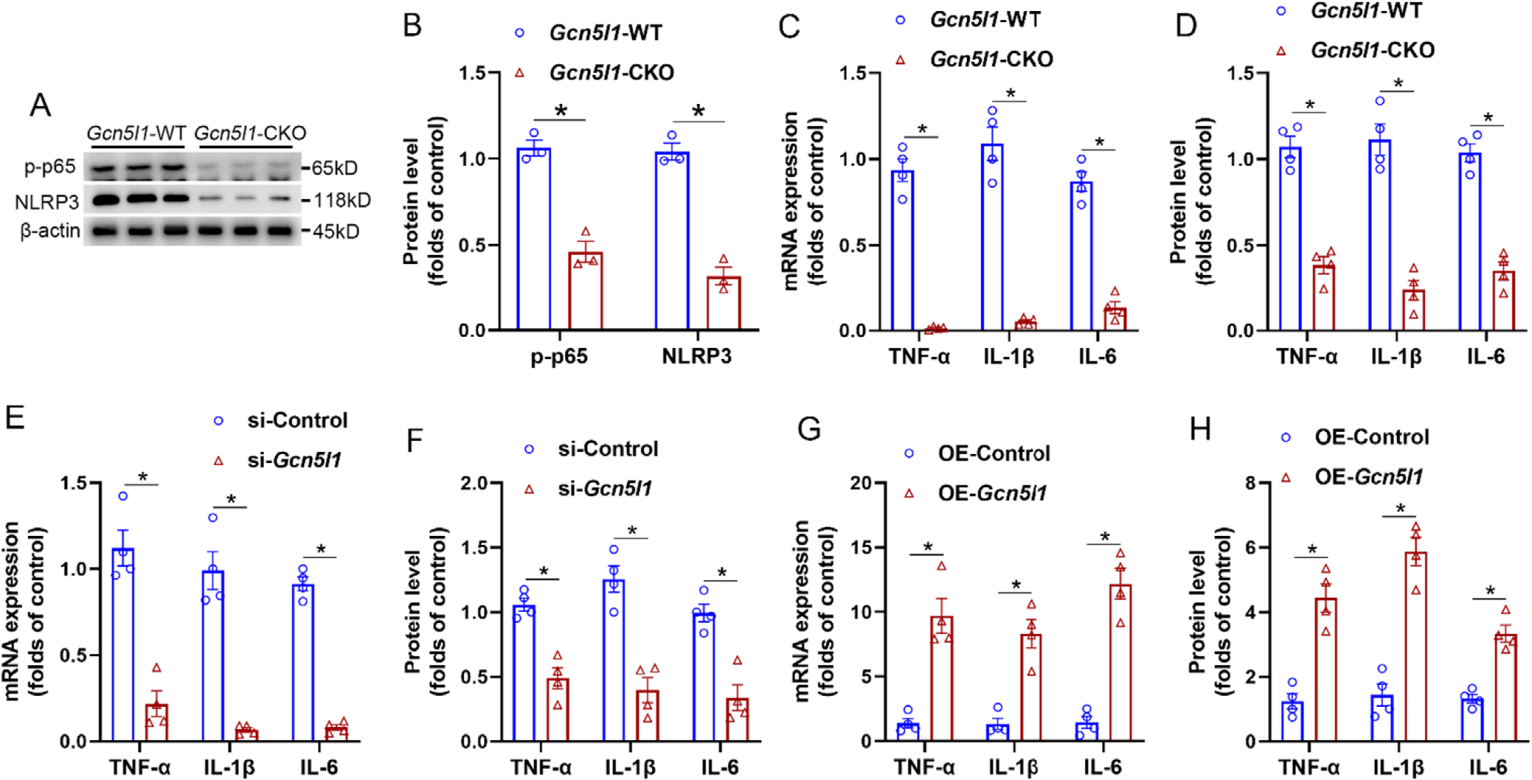
Microglial *Gcn5l1* deficiency or overexpression in vitro regulates HSV‐1‐induced inflammation. (A, B) Western blot analysis (A) and quantification (B) of p‐p65 and NLRP3 protein levels in primary spinal microglia from *Gcn5l1*‐WT and *Gcn5l1*‐CKO mice after PHN. (C, D) The transcription (C) and protein (D) levels of inflammatory cytokines (TNF‐α, IL‐1β, and IL‐6) in primary spinal microglia from *Gcn5l1*‐WT and *Gcn5l1*‐CKO mice after PHN. (E, F) The transcription (E) and protein (F) levels of inflammatory cytokines (TNF‐α, IL‐1β, and IL‐6) in BV‐2 microglial cells with or without *Gcn5l1* knockdown after HSV‐1 infection. (G, H) The transcription (G) and protein (H) levels of inflammatory cytokines (TNF‐α, IL‐1β, and IL‐6) in BV‐2 microglial cells with or without *Gcn5l1* overexpression after HSV‐1 infection. *n* = 4, *p < 0.05.

To further investigate the role of microglial GCN5L1 in HSV‐1‐induced neuroinflammation in vitro, we isolated and cultured primary microglia from the spinal dorsal horn of *Gcn5l1*‐CKO and *Gcn5l1*‐WT mice. We performed western blot analyses to assess key components of microglial activation. Specifically, phosphorylated NF‐κB p65 (p‐p65) was evaluated as a marker of NF‐κB pathway activation, and NLRP3 expression was assessed to reflect inflammasome priming. The results showed that GCN5L1 deficiency significantly reduced the levels of p‐p65 and NLRP3 in primary microglia following HSV‐1 induction (Figure 2A,B), and *Gcn5l1‐deficient* primary microglia exhibited significantly reduced transcription (Figure 2C) and protein (Figure 2D) levels of inflammatory cytokines (TNF‐α, IL‐1β and IL‐6), indicating that GCN5L1 acts upstream of these proinflammatory signalling pathways and contributes to neuroinflammatory amplification through NF‐κB and NLRP3 axis activation. Furthermore, we employed siRNA to knock down *Gcn5l1* expression in microglial BV‐2 cells and also confirmed that *Gcn5l1* knockdown in BV‐2 cells led to a marked decrease in the transcription (Figure 2E) and protein (Figure 2F) levels of inflammatory cytokines (TNF‐α, IL‐1β and IL‐6) after HSV‐1 infection. Conversely, overexpression of *Gcn5l1* via plasmid transfection in BV‐2 cells significantly enhanced the transcription (Figure 2G) and protein (Figure 2H) levels of inflammatory cytokines (TNF‐α, IL‐1β and IL‐6) following HSV‐1 infection. Collectively, these in vitro findings further corroborated the role of microglial GCN5L1 in promoting HSV‐1‐triggered neuroinflammatory responses.

### 3.3 Microglial *Gcn5l1* Deficiency Alleviates Neuroinflammation and Pain Sensitisation by Regulating Mitochondrial Fission–Fusion Homeostasis

**FIGURE 3:**
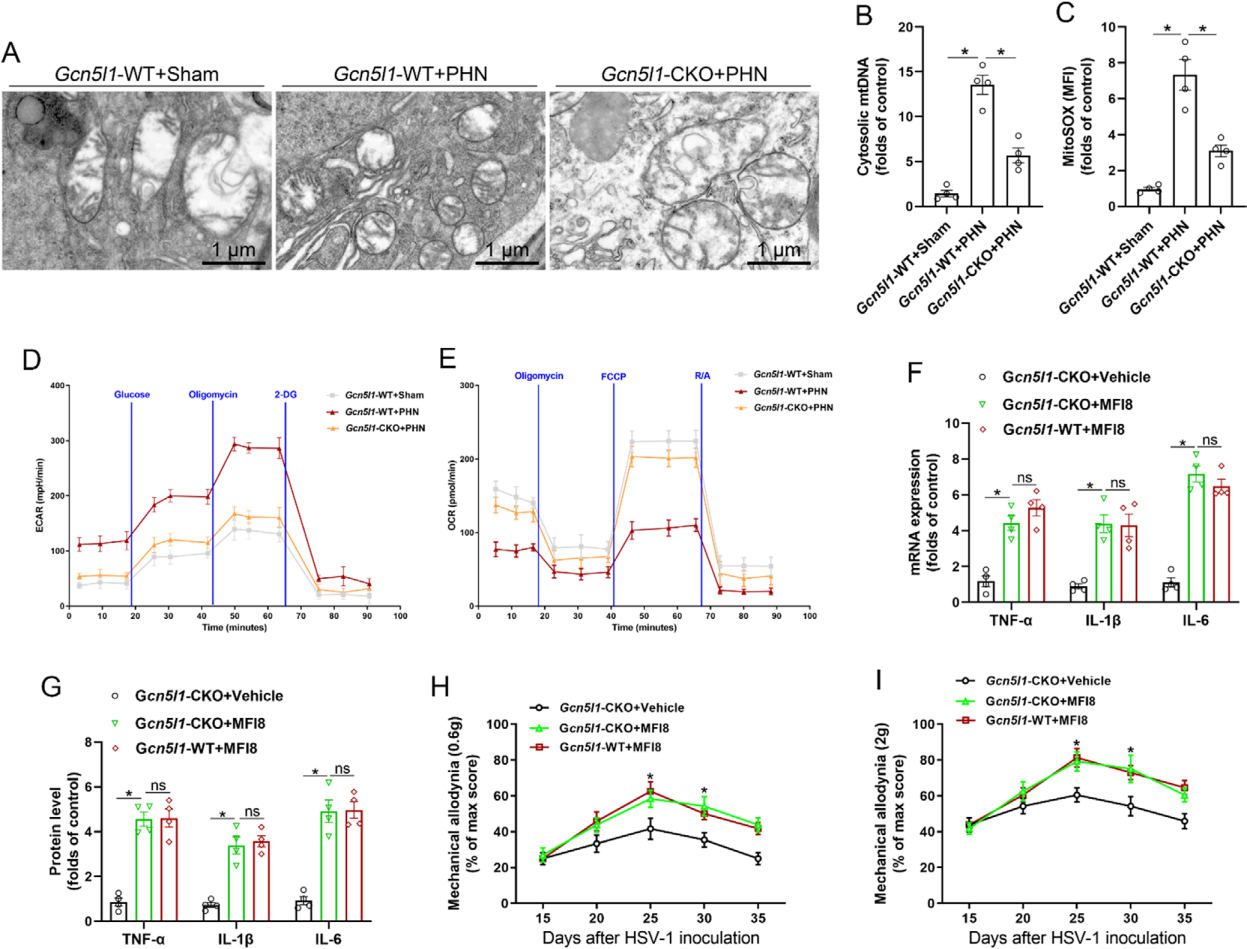
Microglial *Gcn5l1* deficiency alleviates neuroinflammation and pain sensitisation by regulating mitochondrial fission–fusion homeostasis. (A) Transmission electron microscopy of spinal microglia showing mitochondrial fission and fusion changes in *Gcn5l1*‐WT and *Gcn5l1*‐CKO mice with or without PHN. (B, C) Quantification of mtDNA (B) and mtROS (C) levels in microglia of spinal dorsal horn from *Gcn5l1*‐WT and *Gcn5l1*‐CKO mice with or without PHN. (D, E) Seahorse analysis showing ECAR (D) and OCR (E) in microglia of spinal dorsal horn from *Gcn5l1*‐WT and *Gcn5l1*‐CKO mice with or without PHN. (F, G) The transcription (F) and protein (G) levels of inflammatory cytokines (TNF‐α, IL‐1β, and IL‐6) in microglia of spinal dorsal horn from *Gcn5l1*‐WT and *Gcn5l1*‐CKO mice with or without MFI8 intrathecally. (H, I) Mechanical allodynia measured with 0.6 g (H) and 2.0 g (I) von Frey filaments in *Gcn5l1*‐WT and *Gcn5l1*‐CKO mice with or without MFI8 intrathecally. *n* = 4, *p < 0.05.

Given that GCN5L1 is primarily localised in mitochondria and is known to regulate mitochondrial metabolism and homeostasis, we investigated its role in mitochondrial dynamics in microglia during PHN. Transmission electron microscopy revealed that PHN induced increased mitochondrial fission and reduced fusion in microglia from the spinal dorsal horn of wild‐type mice. In contrast, microglial *Gcn5l1* deficiency attenuated mitochondrial fission while promoting fusion in PHN mice (Figure 3A). As excessive mitochondrial fission can lead to cytosolic accumulation of mtDNA and mtROS, both of which contribute to cellular injury and inflammatory responses, we next quantified mtDNA and mtROS levels in microglia from the spinal dorsal horn. We observed that PHN induced markedly elevated mtDNA and mtROS levels in microglia from wild‐type mice, whereas microglial *Gcn5l1* deficiency significantly decreased mtDNA and mtROS levels in the PHN model (Figure 3B,C). To assess the functional consequences on mitochondrial metabolism, we performed Seahorse assays on microglia isolated from the spinal dorsal horn and found that PHN induced elevated ECAR and reduced OCR, indicating impaired mitochondrial oxidative metabolism. In contrast, microglial *Gcn5l1* deficiency significantly decreased ECAR and increased OCR in the PHN model (Figure 3D,E), further supporting the critical role of GCN5L1 in regulating mitochondrial fission–fusion homeostasis.

To further determine whether GCN5L1 promotes neuroinflammation and pain hypersensitivity through disrupting mitochondrial fission–fusion homeostasis, we intrathecally administered MFI8 in PHN mice, which can enhance mitochondrial fission and inhibit fusion. Notably, in the context of microglial *Gcn5l1* deficiency, MFI8 significantly increased the transcription and protein levels of inflammatory cytokines (TNF‐α, IL‐1β and IL‐6). However, in the presence of MFI8 intrathecally in PHN mice, the effects of microglial *Gcn5l1* deficiency on the transcription and protein levels of inflammatory cytokines (TNF‐α, IL‐1β and IL‐6) were abolished (Figure 3F,G). Consistently, MFI8 also markedly aggravated mechanical allodynia to 0.6 and 2 g von Frey filaments in PHN mice with microglial *Gcn5l1* deficiency. In the presence of MFI8 intrathecally in PHN mice, the effects of microglial *Gcn5l1* deficiency on mechanical allodynia were also abolished (Figure 3H,I). These results indicate that microglial *Gcn5l1* deficiency alleviates neuroinflammation and pain sensitisation by regulating mitochondrial fission–fusion homeostasis.

### 3.4 GCN5L1 Regulates Mitochondrial Fission–Fusion Homeostasis and Promotes Inflammation by Acetylating and Activating DRP1

**FIGURE 4:**
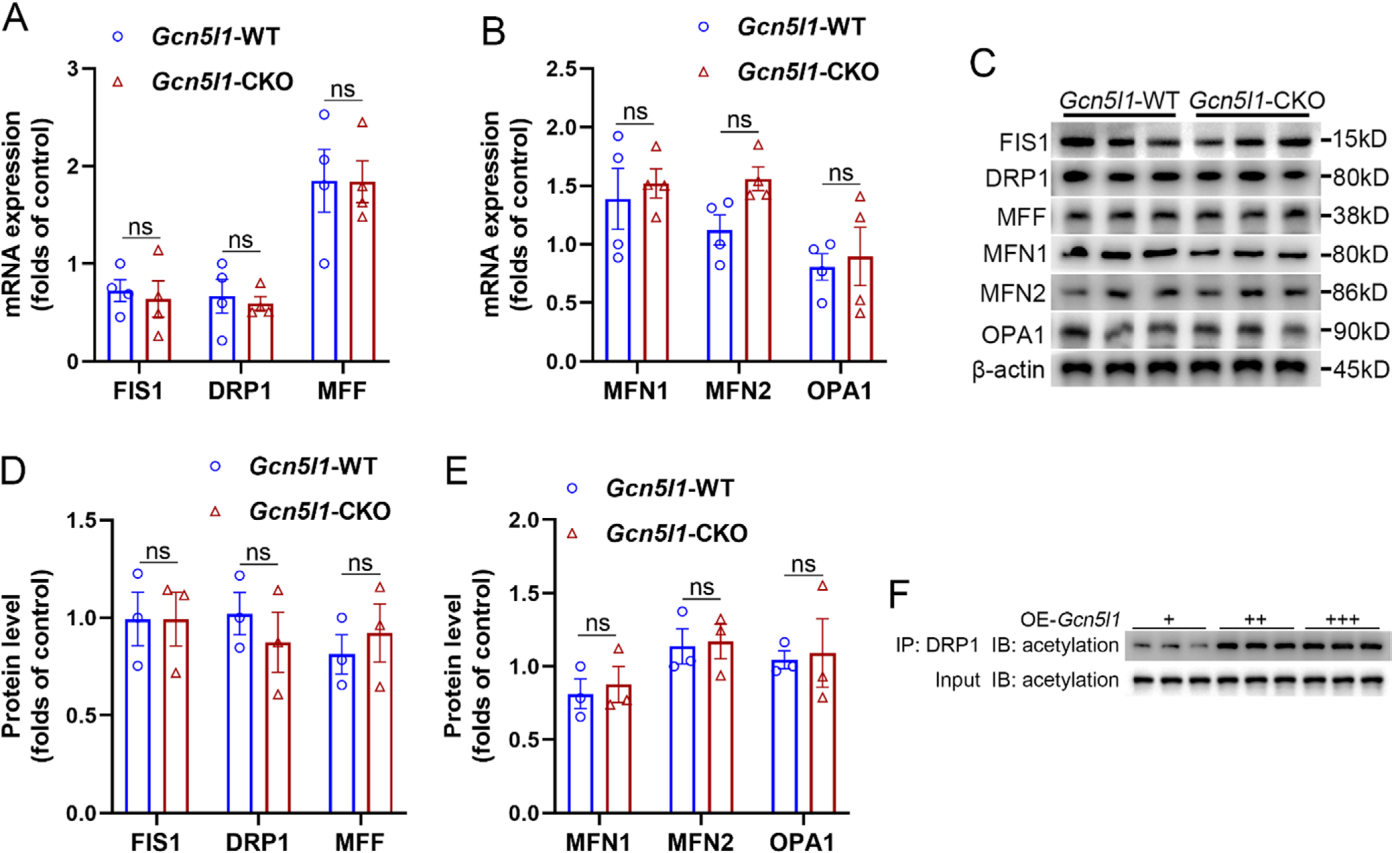
GCN5L1 regulates mitochondrial fission–fusion homeostasis and promotes inflammation by acetylating and activating DRP1. (A) Transcription levels by Q‐PCR of mitochondrial fission‐related genes (FIS1, DRP1, MFF) in microglia of spinal dorsal horn from *Gcn5l1*‐WT and *Gcn5l1*‐CKO mice with PHN. (B) Transcription levels by Q‐PCR of mitochondrial fusion‐related genes (MFN1, MFN2, OPA1) in microglia of spinal dorsal horn from *Gcn5l1*‐WT and *Gcn5l1*‐CKO mice with PHN. (C–E) Western blot analysis (C) and quantification (D, E) of corresponding protein levels in microglia of spinal dorsal horn from *Gcn5l1*‐WT and *Gcn5l1*‐CKO mice with PHN. (F) DRP1 acetylation levels in BV‐2 cells after graded overexpression of *Gcn5l1* by Co‐IP assay. *n* = 4, ns indicates p > 0.05.

Mitochondrial dynamics are tightly controlled by specific regulatory proteins. Mitochondrial fission is primarily mediated by FIS1, DRP1 and MFF, whereas mitochondrial fusion involves MFN1, MFN2 and OPA1. To elucidate the molecular mechanism by which microglial GCN5L1 modulates mitochondrial fission–fusion homeostasis, we examined the expression levels of these core genes. Q‐PCR analysis revealed that the transcription levels of FIS1, DRP1, MFF, MFN1, MFN2 and OPA1 in microglia from the spinal dorsal horn did not differ significantly between wild‐type and microglial *Gcn5l1* deficient mice in the PHN model (Figure 4A,B). Western blotting analysis also showed no substantial differences in the corresponding protein levels between the two groups (Figure 4C–E), suggesting that GCN5L1 does not affect mitochondrial fission–fusion homeostasis through transcriptional or translational regulation of these core proteins. Given that GCN5L1 is a key mitochondrial protein acetylation modulator, we next investigated whether it influenced mitochondrial fission–fusion homeostasis via post‐translational modifications. Co‐IP assays in BV‐2 cells revealed that graded overexpression of *Gcn5l1* led to an increase in DRP1 acetylation levels (Figure 4F). These results suggest that GCN5L1 promotes mitochondrial fission by enhancing the acetylation of DRP1, rather than by altering the overall expression of specific regulatory proteins in mitochondrial fission–fusion.

### 3.5 Inhibiting GCN5L1 Alleviates Neuroinflammation and Pain Sensitisation in PHN

**FIGURE 5:**
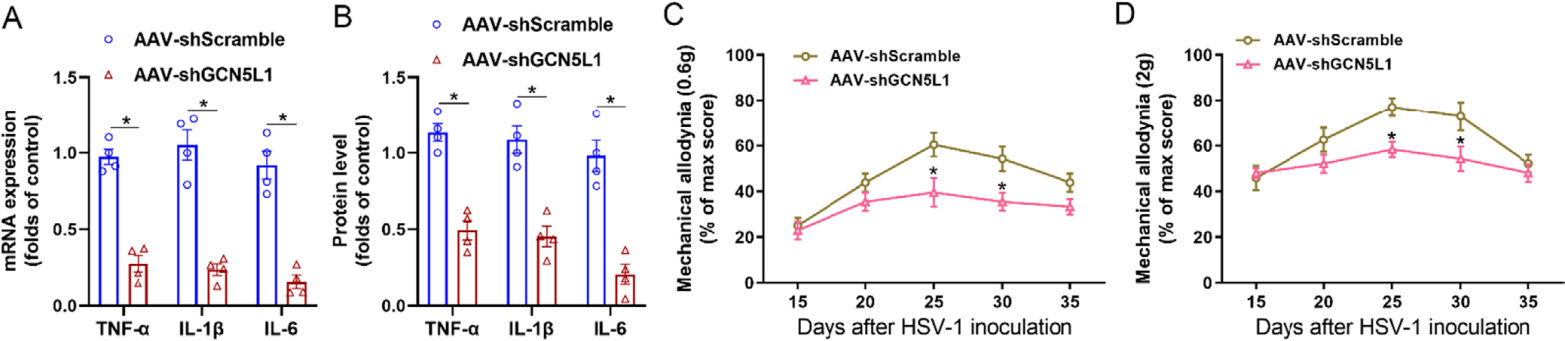
Inhibiting GCN5L1 alleviates neuroinflammation and pain sensitisation in PHN. (A, B) The transcription (A) and protein (B) levels of inflammatory cytokines (TNF‐α, IL‐1β, and IL‐6) in microglia of the spinal dorsal horn from PHN mice treated with AAV‐shGCN5L1 or AAV‐shScramble intrathecally. (C, D) Mechanical allodynia measured with 0.6 g (C) and 2.0 g (D) von Frey filaments in PHN mice treated with AAV‐shGCN5L1 or AAV‐shScramble intrathecally. *n* = 4, *p < 0.05.

Finally, we explored the therapeutic potential of targeting GCN5L1 for PHN by intrathecally delivering an adeno‐associated virus expressing shRNA against Gcn5l1 (AAV‐shGCN5L1). Following PHN induction, intrathecal administration of AAV‐shGCN5L1 led to a significant reduction in the transcription (Figure 5A) and protein (Figure 5B) levels of inflammatory cytokines (TNF‐α, IL‐1β and IL‐6) in the spinal dorsal horn. Moreover, AAV‐shGCN5L1 intrathecally significantly alleviated the mechanical allodynia to 0.6 and 2 g von Frey filaments (Figure 5C,D). These findings indicate that therapeutic inhibition of GCN5L1 effectively reduces neuroinflammation and pain sensitisation in PHN, highlighting its potential as a viable translational target for clinical intervention.

## 4 Discussion

PHN remains a clinically intractable chronic pain condition with limited therapeutic options, largely due to an incomplete understanding of its underlying neuroinflammatory mechanisms. In this study, we identified the mitochondrial acetylation regulator GCN5L1 as a previously unrecognised driver of microglia‐mediated inflammation and pain sensitisation in PHN. We demonstrated that GCN5L1 expression was significantly upregulated in spinal microglia following PHN, and microglial *Gcn5l1* deficiency markedly attenuated inflammatory cytokine production and mechanical allodynia. These findings established GCN5L1 as a critical modulator of neuroinflammation in the context of PHN. Mechanistically, our data revealed that GCN5L1 promoted mitochondrial fission by enhancing the acetylation and activation of DRP1, thereby inducing a shift in mitochondrial dynamics towards fragmentation. This imbalance results in increased mtDNA leakage, elevated mtROS, and profound metabolic reprogramming, all of which are closely associated with proinflammatory microglial phenotypes. Importantly, we provided preclinical evidence that targeting GCN5L1 via intrathecal delivery of AAV‐shGCN5L1 could significantly reduce neuroinflammation and pain hypersensitivity in PHN mice, thereby highlighting the translational relevance of this pathway. Taken together, our findings position GCN5L1 not only as a mechanistic bridge between mitochondrial dynamics and immune activation in microglia, but also as a promising therapeutic target for the treatment of PHN.

A key mechanistic insight from this study is the identification of a GCN5L1‐DRP1‐mitochondrial fission axis as a central driver of microglial inflammatory activation in PHN. We demonstrated that GCN5L1, a mitochondria‐localised acetylation‐related regulator, enhanced the acetylation of DRP1, a critical executor of mitochondrial fission. This post‐translational modification of DRP1 potentiated its activity, thereby promoting excessive mitochondrial fragmentation within spinal microglia following HSV‐1 reactivation [20]. Such pathological mitochondrial fission disrupts organelle homeostasis and leads to leakage of mtDNA and accumulation of mtROS, two well‐established danger signals that fuel innate immune activation [21]. Consistent with this, our data showed that GCN5L1‐driven mitochondrial fragmentation correlated with the activation of the cGAS‐STING pathway, a cytosolic DNA‐sensing mechanism that initiates downstream production of proinflammatory cytokines including TNF‐α, IL‐1β and IL‐6. This cascade not only delineates a molecular route by which mitochondrial stress amplifies inflammatory signalling in microglia, but also exemplifies a broader concept of metabolic‐immune coupling within the central nervous system [22, 23]. By positioning GCN5L1 as a critical upstream regulator of mitochondrial structure and immune response, our findings offer mechanistic clarity to the metabolic reprogramming that underlies microglial proinflammatory phenotypes in chronic neuropathic pain conditions such as PHN.

Beyond its role in regulating mitochondrial dynamics, GCN5L1 also orchestrates a profound shift in microglial metabolic state. Our data demonstrated that elevated GCN5L1 expression in PHN drove a marked increase in ECAR and a concomitant decrease in OCR, indicating a shift toward glycolytic metabolism. This metabolic profile is characteristic of classically activated (M1‐like) microglia, which are known to produce high levels of proinflammatory cytokines and contribute to neuroinflammatory amplification [24]. Such metabolic reprogramming likely reinforces a positive feedback loop between mitochondrial stress and inflammation. Excessive glycolysis not only sustains energy production under mitochondrial dysfunction but also promotes the accumulation of metabolic intermediates that activate innate immune signalling. Thus, GCN5L1‐mediated bioenergetic remodelling may serve as both a consequence and a driver of microglial inflammatory responses, further exacerbating pain sensitisation in PHN. These findings underscore the intimate coupling between metabolic state and immune function in central nervous system pathophysiology [25].

Our study also provides compelling evidence that targeting GCN5L1 offers a viable strategy for alleviating neuroinflammation and pain sensitisation in PHN. Intrathecal delivery of AAV‐shGCN5L1 robustly suppressed the expression of proinflammatory cytokines in the spinal dorsal horn and significantly ameliorated mechanical allodynia in PHN mice. These findings establish a causal link between GCN5L1 activity and disease pathology, and provide preclinical validation of GCN5L1 as a tractable intervention point in neuropathic pain. Compared to other mitochondrial metabolic regulators such as SIRT3 and PGC‐1α, which broadly modulate mitochondrial homeostasis and energy metabolism, GCN5L1 exerts a distinct and specific function through post‐translational acetylation of mitochondrial proteins [26, 27]. In particular, its role in modulating DRP1 acetylation positions it as a nodal regulator of mitochondrial dynamics, offering therapeutic specificity that could reduce off‐target effects associated with more global metabolic modulators. Nonetheless, translational application of GCN5L1 inhibition faces notable challenges. The development of selective small‐molecule inhibitors that effectively target GCN5L1's mitochondrial activity without impairing global acetylation processes remains a key hurdle. Additionally, ensuring the delivery of such compounds across the blood‐spinal cord barrier and into microglia adds another layer of complexity. Future efforts should focus on high‐throughput screening of GCN5L1‐binding compounds, structural characterisation of its acetyltransferase domain, and in vivo pharmacodynamic studies to advance GCN5L1‐targeted therapies from bench to bedside [28, 29].

While our findings provide compelling evidence for a central role of GCN5L1 in regulating microglial mitochondrial dynamics and neuroinflammation in PHN, several limitations should be acknowledged. First, the present study is primarily based on murine models and in vitro microglial systems. The clinical relevance of GCN5L1 expression and function in human PHN remains to be validated in patient‐derived spinal tissue or cerebrospinal fluid samples. Second, although we identified DRP1 as a downstream effector of GCN5L1‐mediated acetylation, the specific lysine residues involved and the structural mechanisms by which acetylation enhances DRP1 activity require further elucidation. Future studies should also investigate whether GCN5L1 regulates the acetylation of additional mitochondrial proteins involved in inflammatory signalling or metabolic control. Such insights may reveal broader roles for GCN5L1 in immunometabolic remodelling beyond DRP1‐dependent fission. Moreover, it remains to be determined whether a similar GCN5L1‐DRP1 axis contributes to microglial dysfunction in other neurological conditions characterised by chronic inflammation such as Alzheimer's disease, multiple sclerosis or neuropathic pain of different etiologies. Addressing these questions will be essential for establishing GCN5L1 as a generalisable target for neuroimmune modulation [30, 31, 32].

Together, our findings uncover a previously unrecognised role of GCN5L1 in orchestrating mitochondrial fission, metabolic reprogramming and microglial‐driven neuroinflammation in PHN. By establishing the GCN5L1–DRP1 axis as a key molecular pathway linking mitochondrial dysfunction to chronic pain pathology, this study not only advances our understanding of the immunometabolic basis of PHN, but also provides a rationale for targeting mitochondrial acetylation machinery as a novel therapeutic avenue in neuroinflammatory disorders.

## Author Contributions

**Wang Li:** writing – original draft (equal). **Xin Cao:** methodology (equal). **Shenghan Wang:** visualization (equal). **Xuedong Jin:** data curation (equal). **Hongqian Wang:** conceptualization (equal), writing – review and editing (equal).

## Conflicts of Interest

The authors declare no conflicts of interest.

## Data Availability

The data that support the findings of this study are available from the corresponding author upon reasonable request.
